# Cheminformatics Microservice V3: a web portal for chemical structure manipulation and analysis

**DOI:** 10.1186/s13321-025-01094-1

**Published:** 2025-09-23

**Authors:** Kohulan Rajan, Venkata Chandrasekhar, Nisha Sharma, Sri Ram Sagar Kanakam, Felix Baensch, Christoph Steinbeck

**Affiliations:** 1 Institute for Inorganic and Analytical Chemistry, Friedrich Schiller University Jena, Lessingstr. 8, 07743 Jena, Germany; 2 Beilstein-Institut Zur Förderung Der Chemischen Wissenschaften, Trakehner Straße 7-9, 60487 Frankfurt Am Main, Germany

**Keywords:** CDK, RDKit, Open Babel, Cheminformatics, Toolkits, Microservice, Web application

## Abstract

The widespread adoption of open-source cheminformatics toolkits remains constrained by technical implementation barriers, including complex installation procedures, dependency management, and integration challenges. Here, we present *Cheminformatics Microservice V3*, a significant update to the existing platform that provides unified programmatic access to cheminformatics libraries, including RDKit, Chemistry Development Kit (CDK), and Open Babel through a RESTful API framework. This latest version features a newly developed, interactive web-based frontend built with React, providing users with an intuitive graphical interface for manipulating and analysing chemical structures. The frontend supports essential cheminformatics operations, including structure editing, PubChem database integration, batch molecular processing, and standardised InChI/RInChI identifier generation. The microservice V3 addresses critical accessibility barriers in computational chemistry by providing researchers with immediate access to analytical tools, eliminating the need for specialised technical expertise or complex software installations. This approach facilitates reproducible research workflows and broadens the utilisation of cheminformatics methodologies across interdisciplinary research communities. The platform is publicly accessible at https://app.naturalproducts.net, and the complete source code and documentation are available on GitHub.

## Scientific contribution

*Cheminformatics Microservice V3* aims to provide easily accessible and reproducible cheminformatics tools. This latest version introduces a unified, web-based interface that significantly enhances user accessibility to open-source cheminformatics toolkits. By making the web application and API endpoints freely available, along with fully open and well-documented source code, we aim to eliminate barriers to use, encourage community-driven development, and foster an environment of collaboration and innovation.

**Graphical Abstract:**

## Graphical abstract

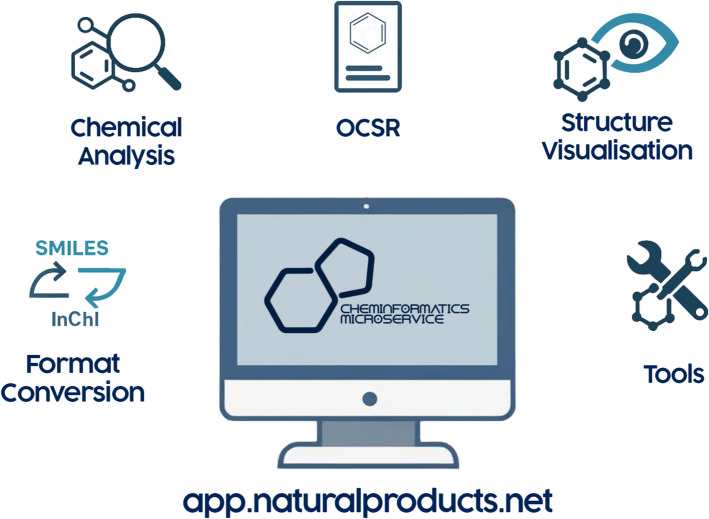

## Introduction

Over the last three decades, cheminformatics has experienced substantial progress, largely driven by the development of numerous open-source software toolkits [1, 2]. While these toolkits provide essential functionalities, researchers often face practical barriers when integrating multiple tools into their workflows. These include complex installation procedures, dependency and compatibility issues, deployment overheads, and the need for programming expertise. The fragmentation of chemical toolkits across multiple programming languages and platforms can complicate the development of integrated research workflows. To address these limitations and streamline access to cheminformatics functionalities, the Cheminformatics Microservice [3] provides a unified platform that consolidates access to widely-used toolkits including RDKit [4], Chemistry Development Kit (CDK) [5, 6], and Open Babel [7]. The platform exposes core functionality via a standardised RESTful API, enabling researchers to access essential cheminformatics operations through a consistent interface.

The initial version of this was developed using FastAPI, a Python web framework. The Cheminformatics Microservice provided a robust and efficient RESTful API interface with the application being containerized using Docker, incorporating all required dependencies and versioned cheminformatics toolkits. Tools such as chemical structure generation [8], sugar removal [9] and Optical Chemical Structure Recognition (OCSR) through Deep lEarning for Chemical ImagE Recognition (DECIMER) [10] were integrated within a single, portable deployment unit—ensuring reproducibility, platform independence, and consistent execution across environments. To lower adoption barriers and support reproducibility, the complete, versioned source code was made openly available on GitHub, along with comprehensive documentation. A publicly accessible instance was hosted at https://api.naturalproducts.net, and pre-built Docker images were provided via Docker Hub, enabling rapid testing, integration, and extension within diverse computational environments.

While the microservice architecture enabled programmatic access to toolkit functionalities, it still required users to possess technical expertise to interact with Application Programming Interfaces (APIs) endpoints and integrate the results into their workflows [11, 12]. To further improve the accessibility and usability of cheminformatics resources, a user-friendly frontend interface is essential. Web-based scientific applications have gained popularity due to their accessibility, platform independence, and ability to leverage modern visualisation technologies [13, 14]. In this work, we present *Cheminformatics Microservice V3*, a significant update that introduces a fully designed frontend developed using React [16] and accessible at https://app.naturalproducts.net.

In addition to the frontend, this version introduces several backend components, including functional groups detection, filtering mechanisms and substructure highlighting functionality, alongside software optimisations. The frontend also includes PubChem [15] search capabilities, a chemical structure editor for drawing and editing molecular structures, support for batch depiction of up to 50 molecules, and functionalities for generating InChI [16] and RInChI [17] representations.

The development of *Cheminformatics Microservice V3* adheres to established research data management practices, with a focus on enabling reproducibility, which is central to the FAIR (Findable, Accessible, Interoperable, and Reusable) principles of research [18]. This release enhances both the accessibility and functionality of cheminformatics tools, enabling researchers to handle, process, and analyse chemical data with minimal technical barriers, while maintaining full backwards compatibility. To support open science and broad community adoption, the complete software stack, including source code and deployment resources, is freely available at: https://github.com/Steinbeck-Lab/cheminformatics-microservice.

### Implementation

#### Technical Architecture

The backend of *Cheminformatics Microservice V3* retains the stable and modular architecture introduced in the initial release. It is implemented in Python and uses the FastAPI framework [19] to expose cheminformatics functionalities through a RESTful API. Toolkit integration is achieved using a hybrid approach: Python-native libraries, such as RDKit and Open Babel, are incorporated directly, while Java-based tools—including the Chemistry Development Kit (CDK), Sugar Removal Utility (SRU), and OPSIN [20]—are accessed using JPype [21], a Python bridge to Java. The system is containerized using Docker, ensuring reproducibility, consistent deployment across environments, and simplified dependency management. This architecture provides a robust foundation for cheminformatics workflows, allowing for future extensions without disrupting existing services.

**Fig. 1:**
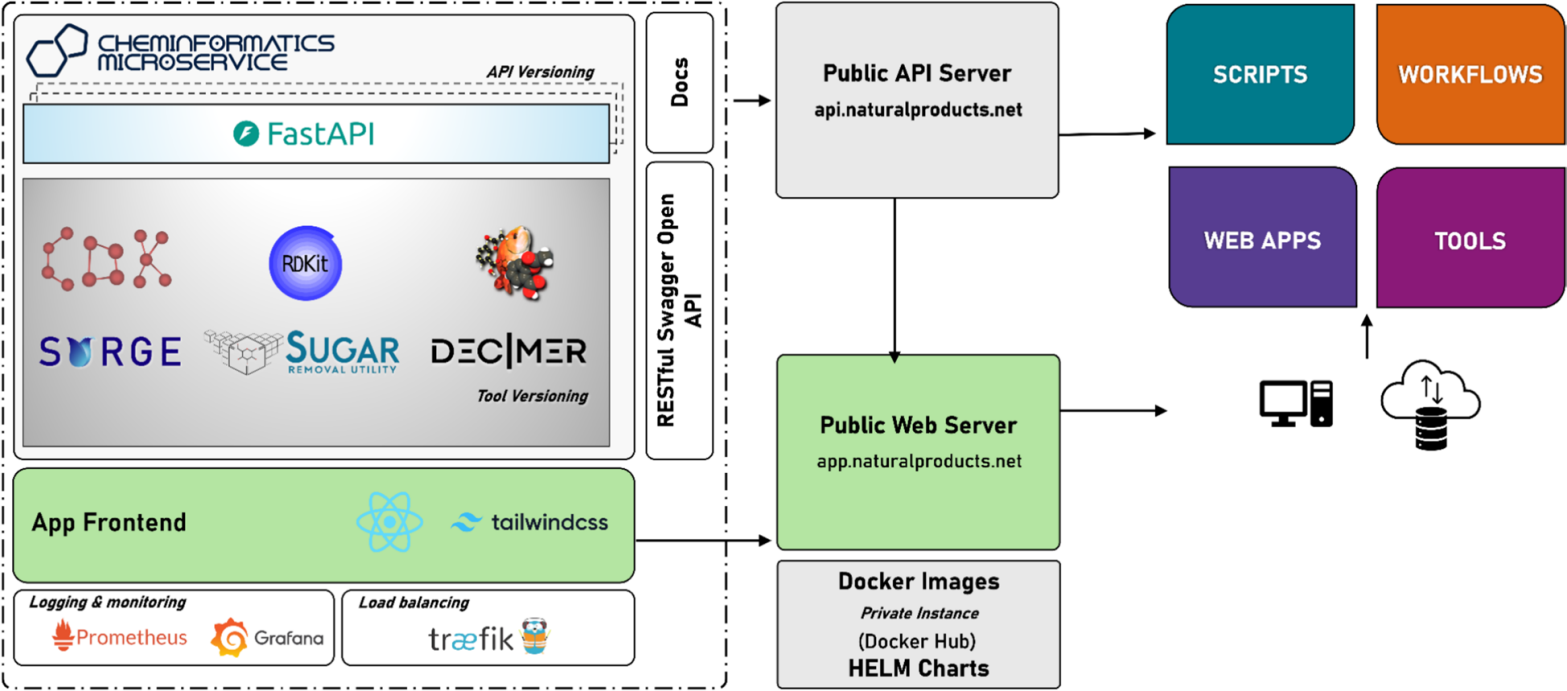
The Cheminformatics Microservice V3 public server architecture

The frontend of *Cheminformatics Microservice V3* was developed using modern web technologies, with React chosen as the core framework due to its strong community support, extensive ecosystem of reusable components, and widespread adoption in scientific and enterprise software development. This decision was guided not only by technical considerations—such as modular architecture, reusability, and ease of maintenance—but also by a broader commitment to software sustainability. Adopting a widely supported and well-documented framework like React lowers the entry barrier for external contributors, thereby facilitating community-driven development. The implementation features a component-based architecture with modular service layers, custom hooks for handling chemical data, and a responsive UI design built with Tailwind CSS [22]. The frontend (web interface) of the microservice interacts with the backend via RESTful API endpoints, with HTTP requests managed through the Axios [23] library. This communication is abstracted through a dedicated API service layer, encapsulating all interactions with the backend, which also provides separation of concerns and enhances maintainability. This ensures that future updates or additions to the API can be implemented with minimal disruption to the existing codebase. The application is organised into functionally specialised pages, each containing related tools and components, with the frontend at https://app.naturalproducts.net/. Figure 1 illustrates the new frontend and backend architecture. The backend API endpoints remain unchanged and can be used as before.

#### Features

*Cheminformatics Microservice V3* enhances backend functionality while maintaining the existing five-module architecture, comprising *chem*, *convert*, *depict*, *ocsr*, and *tools*. Three new tools expand the platform’s reach: (i) an Ertl functional‑group finder [24]; (ii) a unified filter suite covering Pan-Assay Interference compounds (PAINS) [25], Lipinski’s rule of five [26], Veber[27], Rapid Elimination Of Swill (REOS) [28], Ghose [29] and Rule‑of‑3 [30], along with Quantitative Estimation of Drug-likeness (QED) [31], Synthetic Accessibility (SA) score[32] and Natural Product (NP) likeness [33] metrics; and (iii) a PubChem search that retrieves molecular structures for downstream use [34]. Format conversion now supports batch processing. For workflows that do not require OCSR, a lightweight Docker image focused solely on cheminformatics tasks is also available under the tag—latest-lite. The *depict* module now offers finer control over 2‑D rendering, including rotation and substructure highlighting.

The newly introduced frontend provides an interactive web-based graphical interface, enabling intuitive access to all backend functionalities. Notable frontend features include:

- A Structure Explorer facilitating molecule queries and retrieval directly from PubChem, with integrated 2D and 3D visualisation capabilities.
- Generation of InChI and RInChI identifiers using the official InChI Trust implementation.
- An integrated structure editor utilising Ketcher 3.0 [35], allowing chemical structure creation and modification.
- Batch conversion and depiction functionality, accommodating simultaneous visualisation of up to 50 molecules via CDK or RDKit, with support for substructure highlighting and CIP rule integration.

All input fields feature illustrative examples, and each result field is equipped with convenient copy and download options, facilitating straightforward data handling and reuse. Best practices for sustainable software development were followed throughout the development and maintenance of this work to ensure reproducibility. Clear and consistent version control was maintained, and the versions of all tools and packages used have been thoroughly documented. All API endpoints have been optimised for better performance and updated to support the latest releases of the underlying toolkits, tools, and environment dependencies.

The standard distribution ships with built-in DevOps tooling: Prometheus gathers metrics and Grafana provides real‑time dashboards for resource and performance monitoring. Production Docker Compose YAML configuration files include the same monitoring stack. Front‑end activity can be assessed with Matomo[36], which offers IP randomisation and automatic log purging to protect user privacy. Recent‑search terms are stored solely in the browser’s local storage; no user‑submitted data is retained on the server.

The publicly accessible instance of the Cheminformatics Microservice is built and released through CI/CD workflows driven by GitHub Actions. We have achieved over 90% test coverage, and both production and development deployments are fully automated.

## Results and discussion

The new *Cheminformatics Miroservice V3* significantly improves upon the previous version by providing performance optimisation, more API endpoints and a comprehensive user interface that makes the cheminformatics toolkits more accessible to researchers without any prior programming experience. Building on the cheminformatics toolkits integrated in the initial release, RDKit, CDK, and OpenBabel, as well as tools including *Surge*, the Sugar Removal Utility, and the DECIMER (Deep Learning for Chemical ImagE Recognition) OCSR engine, the current version introduces several more features and enhancements. These include the integration of the Ketcher structure editor for molecular structure drawing, a PubChem search and retrieval feature utilising the Power User Gateway (PUG) REST API [34], and expanded functionality for InChI and RInChI generation and processing. The current version extends existing features while maintaining backwards compatibility, enabling established users to continue utilising familiar tools seamlessly. At the same time, the improved interface and enhanced functionality lower the barrier to entry for new users, facilitating easier handling and processing of chemical structure data.

The frontend application could be accessed via our public instance https://app.naturalproducts.net, and the backend service via https://api.naturalproducts.net.

**Fig. 2:**
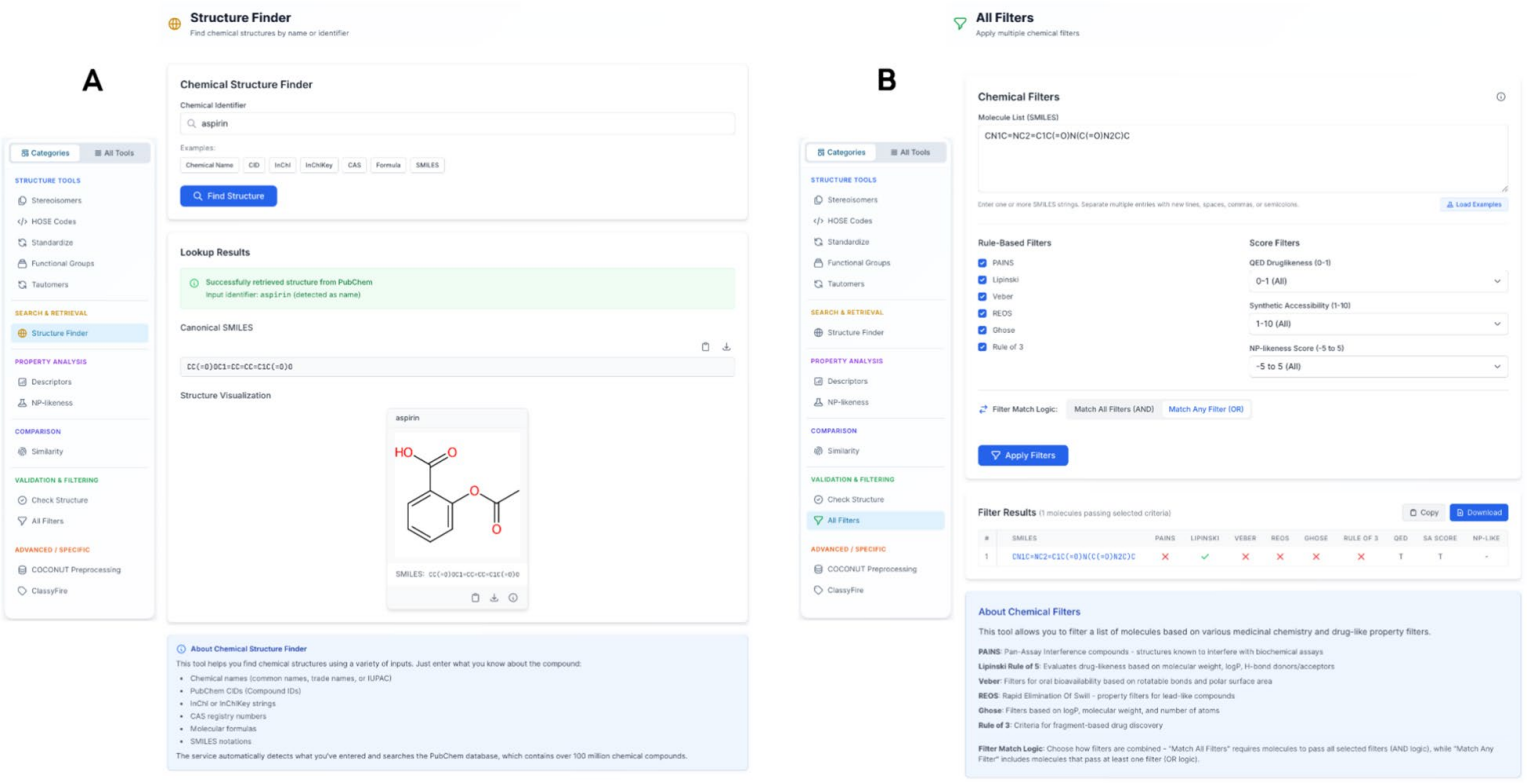
Structure Finder using PubChem [**A**] and Chemical Filters [**B**] with information about them displayed below

The interface of the frontend is organised into five main tabs, providing users with streamlined access to the microservice's functionalities. The Chemical Analysis section integrates all modules related to structure manipulation, standardisation, and descriptor calculation. It also includes all functionalities from the *chem* endpoint of the microservice, in addition to newly introduced modules such as the Structure Finder and All Filters, as shown in Fig. 2.

The Format Conversion section provides modules for converting SMILES [37] representations into various other chemical string formats, as well as for generating 2D and 3D molecular coordinates via the *convert* endpoint. The frontend also offers functionality that allows users to easily copy or download the converted outputs.

**Fig. 3:**
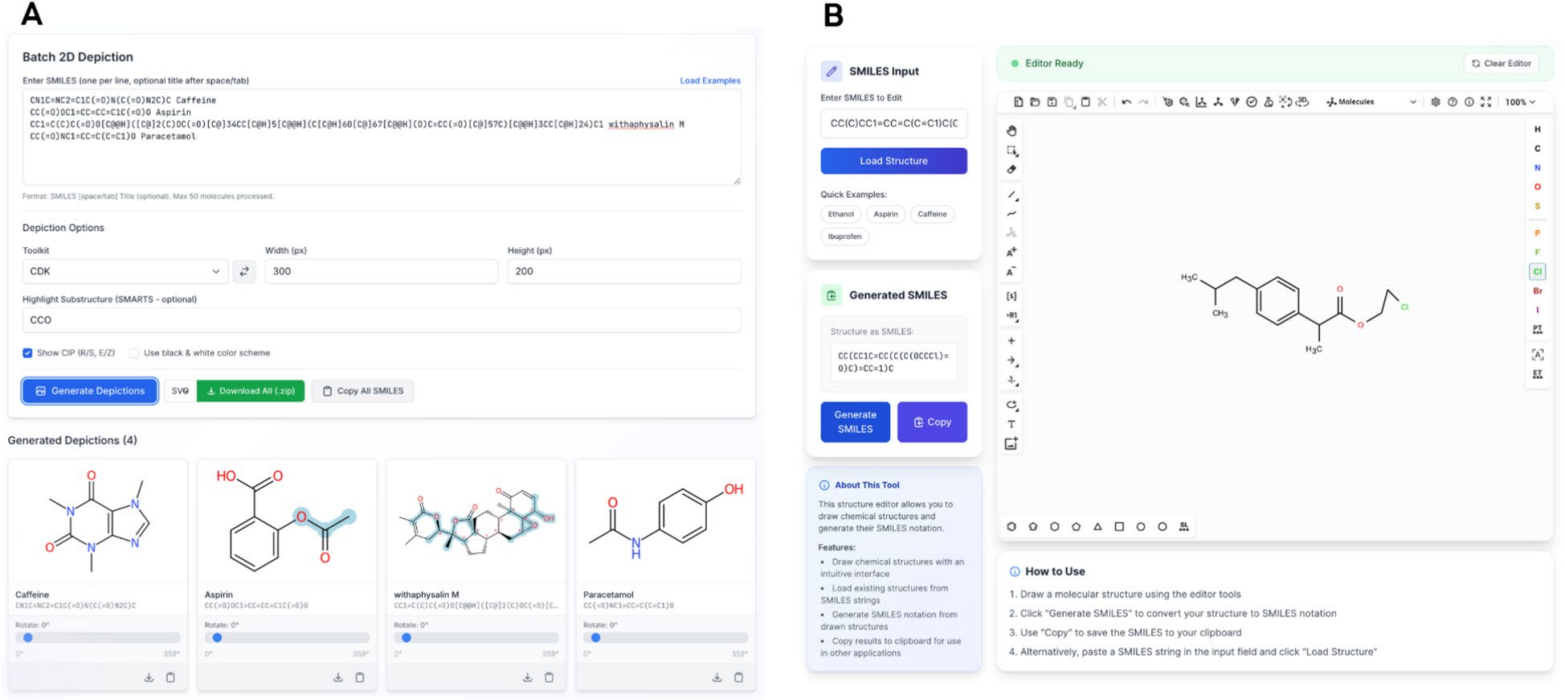
Batch 2D depiction of molecules using CDK with CIP stereochemical annotations and substructure highlighting [**A**]. The Ketcher molecular editor interface is part of the "Draw a Structure" [**B**]

The Depiction section enables users to generate 2D depictions of up to 50 molecules simultaneously using CDK or RDKit via the *depict* endpoint. The resulting images are rendered in SVG format and can be downloaded as a zipped archive or individually. When using CDK for 2D depiction, stereochemical annotations based on the Cahn–Ingold–Prelog [38] (CIP) priority rules can be applied. The 2D depiction endpoint has been improved to support substructure highlighting. For 3D representations, users can generate molecular structures from computed coordinates using either RDKit or Open Babel, with adjustable configurations. These 3D structures are rendered via JSmol, allowing users to interact with the molecules and capture desired poses using a built-in screenshot feature. The Structure Explorer feature allows retrieval of molecules from PubChem using identifiers such as names, CIDs, SMILES strings, or molecular formulas, and supports visualisation in both 2D and 3D formats. The Draw a Structure feature provides users the option to load and edit a molecular structure from SMILES or draw a structure from scratch using the Ketcher molecular editor; the final structure can then be exported as a SMILES string, In Fig. 3, users can see the depicted chemical structures alongside the name, with substructures highlighted and the “Draw a Structure” panel which shows a modified version of a molecule edited using the integrated molecular editor.

The Tools section includes tools such as the Sugar removal utility and Surge structure generator via the *tools* endpoint. This now includes the newly implemented IUPAC International Chemical Identifier (InChI) [39] and Reaction InChI (RInChI) [17] converters that are integrated with the Ketcher structure editor. This allows generating InChIs and RInChIs directly from the molecular structures and chemical reactions drawn within the web interface. These implemented functionalities are analogous to the InChI web demo [40].

The OCSR section provides access to the DECIMER toolkit via the *ocsr* endpoint, which facilitates the identification, segmentation, and translation of chemical structure images into a machine-readable representation. The predicted structures are also rendered as 2D depictions for immediate visual verification.

The frontend implementation prioritises user accessibility and usability through responsive UX/UI design and compatibility with various screen sizes from desktop workstations to tablets and mobile devices, ensuring a consistent and intuitive experience across platforms. Support for dark mode helps reduce eye strain during extended usage. Clear information boxes, error messages and loading indicators enhance the user experience by providing immediate and informative feedback during processing-intensive operations.

### InChI and RInChI implementation and features

The IUPAC International Chemical Identifier (InChI) [16] is a textual identifier for chemical substances. Due to its unique and canonical line notation, it is possible to search for chemical structures both within large databases and on the internet. The Reaction InChI (RInChI) [17] is an extension of the InChI concept, whereby the components of reactions are combined to generate a unique identifier.

The implementation of a web-based tool that combines a structural editor like Ketcher with the functionalities of the InChI and RInChI libraries allows users to interactively generate and analyse the InChIs and RInChIs directly from the chemical structure input. Ketcher provides a mol file for the drawn structure, which is then used as the input for the InChI functionalities. The InChI library utilises this mol file to generate an InChI, which is subsequently returned along with its InChIKey, the Auxiliary Information (AuxInfo) [41], and, if applicable, the log statements. The RInChI library will utilise the RXN file provided by Ketcher as the input format to calculate the RInChI and its keys and RAuxInfo.

All applicable InChI options can be selected via checkboxes or drop-down menus. As with the InChI Web Demo [40], it is possible to alternate between different versions of InChI for the intended calculation. In addition to the current version 1.07.3, the previous version 1.06 and the prototype for treating molecular inorganics are also available.

The features offered by the frontend and backend can be further extended using open cheminformatics toolkits, either by leveraging existing wrappers or developing new ones. As outlined in our original work, achieving granular control over these newly implemented modules ensures that the software package remains stable and maintainable. All implementations are thoroughly documented, and the full documentation is available at: https://api.naturalproducts.net/latest/docs.

## Conclusion

The *Cheminformatics Microservice V3* builds upon our previously published work by pairing its modular microservice architecture with a user-friendly graphical web interface, making cheminformatics tools readily available to researchers regardless of their access to specialised software or programming expertise.

The Cheminformatics Microservice platform offers a unified architecture that integrates multiple open-source cheminformatics toolkits, reducing the need for users to manage individual software environments. The intuitive frontend enables interactive visualisation and manipulation of molecular structures across both desktop and mobile devices. At the same time, the backend API continues to streamline workflows by consolidating visualisation, analysis, and conversion tasks within a single environment.

Our platform’s modular architecture and containerized deployment ensure reproducibility, effortless maintenance and extensibility. This design facilitates the integration of new cheminformatics tools as they emerge and enables adaptation to evolving user needs. The platform adheres to software development best practices, including standardised code contribution protocols, semantic versioning, bi-annual updates, and continuous integration/deployment pipelines via GitHub Actions.

By releasing both the source code and documentation as fully open and publicly accessible, the Cheminformatics Microservice aims to further facilitate access to cheminformatics library functionalities. Its emphasis on reproducibility and extensibility makes it a valuable tool for researchers pursuing data-driven chemical analysis and collaborative projects.

## Data Availability

Project name: Cheminformatics Microservice & UI. Project home page: https://github.com/Steinbeck-Lab/cheminformatics-microservice. Docker Image: https://hub.docker.com/r/nfdi4chem/cheminformatics-microservice. Live instance: https://app.naturalproducts.net. Operating system: Platform independent (web-based). Programming language: JavaScript (Frontend), Python (Backend API). Other requirements: Modern web browser with JavaScript enabled. License: MIT License. Current version: v3.4.0. DOI of archived current release: update??. Documentation: Home page: https://docs.api.naturalproducts.net/ API: https://api.naturalproducts.net/latest/docs: Python Documentation https://cheminformatics-microservice.readthedocs.io/en/latest/ Any restrictions on use by non-academics: None

## Abbreviations

API: Application programming interfaces
CDK: Chemistry development kit
CI/CD: Continuous integration and continuous deployment
CIP: Cahn–Ingold–Prelog
CPU: Central processing unit
CSS: Cascading style sheets
DECIMER: Deep lEarning for Chemical ImagE Recognition
FAIR: Findable, accessible, interoperable, and reusable
HTTP: Hypertext transfer protocol
InChI: IUPAC international chemical identifier
IUPAC: International union of pure and applied chemistry
NP: Natural product
OCSR: Optical chemical structure recognition
OPSIN: Open parser for systematic IUPAC nomenclature
PAINS: Pan-assay interference compounds
PUG: Power user gateway
QED: Quantitative estimation of drug-likeness
RAM: Random access memory
RDM: Research data management
REOS: Rapid elimination of swill
RInChI: Reaction InChI
REST: Representational state transfer
SA: Synthetic accessibility
SMILES: Simplified molecular-input line-entry system
SVG: Scalable vector graphics
UI: User interface
UX: User experience
VM: Virtual machine
YAML: Yet another markup language
